# Nucleotide Pool Imbalance and Antibody Gene Diversification

**DOI:** 10.3390/vaccines9101050

**Published:** 2021-09-22

**Authors:** Asim Azhar, Nasim A. Begum, Afzal Husain

**Affiliations:** 1 Department of Biotechnology, Faculty of Life Sciences, Aligarh Muslim University, Aligarh 202002, India; azharasim@gmail.com; 2 Department of Immunology and Genomic Medicine, Graduate School of Medicine, Kyoto University, Kyoto 606-8501, Japan; nasim@mfour.med.kyoto-u.ac.jp; 3 Department of Biochemistry, Faculty of Life Sciences, Aligarh Muslim University, Aligarh 202002, India

**Keywords:** dNTP, V(D)J recombination, SHM, CSR, antibody, SAMHD1

## Abstract

The availability and adequate balance of deoxyribonucleoside triphosphate (dNTP) is an important determinant of both the fidelity and the processivity of DNA polymerases. Therefore, maintaining an optimal balance of the dNTP pool is critical for genomic stability in replicating and quiescent cells. Since DNA synthesis is required not only in genomic replication but also in DNA damage repair and recombination, the abnormalities in the dNTP pool affect a wide range of chromosomal activities. The generation of antibody diversity relies on antigen-independent V(D)J recombination, as well as antigen-dependent somatic hypermutation and class switch recombination. These processes involve diverse sets of DNA polymerases, which are affected by the dNTP pool imbalances. This review discusses the role of the optimal dNTP pool balance in the diversification of antibody encoding genes.

## 1. Introduction

Immunoglobulins (Ig) are proteins expressed by B lymphocytes in response to pathogens, vaccine candidates, or artificial synthetic antigens. An Ig is composed of two heavy (IgH) and two light (IgL) chains. In humans, IgH chains are encoded by heavy chain locus present on chromosome 14, whereas light chains are encoded by either Igκ or Igλ chain loci that are present on chromosome 2 and chromosome 22, respectively. There are five isotypes for IgH chain (Igμ, Igδ, Igγ, Igε, and Igα), whereas the IgL chain has only two types (Igκ and Igλ). The IgG and IgA isotypes are further divided into four (Igγ1, Igγ2, Igγ3, and Igγ4) and two (Igα1 and Igα2) subtypes, respectively [1]. The N-terminal regions of heavy and light chains show high sequence variability and constitute the variable (V) region of the antibody. The V region of the IgH chain is formed by the joining of V_H_ (variable), D_H_ (diversity), and J_H_ (joining) gene segments, whereas the V region of the IgL chain is formed by the joining of only V_L_ and J_L_ segments [2,3]. The heavy chain constant (C_H_) regions of IgM, IgD, IgG, IgE, and IgA are encoded by Cμ, Cδ, Cγ, Cε, and Cα exon clusters, respectively, whereas light chains constant (C_L_) regions, are encoded by constant region exons of Igκ and Igλ loci [4].

The generation of effective antibodies by lymphocytes requires both antigen-independent and antigen-dependent diversification of Ig genes during the maturation of B lymphocytes [1]. Antigen-independent rearrangements of germline V, D, and J segments of the Ig genes generate the primary antibody repertoire. The diversity of this repertoire is contributed by both the combinatorial diversity that results from the choices of germline V, D, and J gene segments and junctional diversity, which arises as a result of imprecise end-joining of germline segments [1]. However, the antibody repertoire produced by V(D)J recombination alone is not diverse enough to generate high-affinity antibodies against a wide range of antigenic challenges that an individual may encounter in a lifetime. Therefore, the repertoire of B-lymphocytes is further diversified following their encounter with antigens. The antigen activation of B lymphocytes induces the expression of activation-induced cytidine deaminase (AID), an enzyme that is responsible for somatic hypermutation (SHM) and class switch recombination (CSR) of the immunoglobulin genes [5,6]. The processes of CSR and SHM engrave antibody memory, a critical requirement for humoral immunity, into the immunoglobulin genes [7].

The availability and adequate balance of deoxyribonucleoside triphosphates (dNTPs) are critical not only for DNA replication but also for DNA repair, and recombination [8,9,10]. The imbalances in the intracellular concentrations of dNTPs outside their normal ranges are closely associated with genomic instability, an inherent property of most human cancers. The dNTP pool imbalance affects genomic stability by affecting the rate of DNA synthesis and its fidelity during DNA replication, DNA repair, and recombination [8,9,10,11,12]. The DNA in our cell is subjected to various mechanical, chemical, and physical stress, resulting in the point mutations and formation of single- or double-strand DNA breaks (SSB or DSB). In addition, DNA breaks are also formed during physiological processes such as meiotic recombination, rearrangement of immunoglobulin gene segments, transposition of mobile elements, and integration of viral DNA into our genome [13]. As DNA repair involves various DNA polymerases, the dNTP pool imbalance affects DNA damage, repair, and mutagenesis. In this review, we specifically discuss the role of the optimal dNTP pool in the somatic hypermutation and recombination of antibody encoding genes.

## 2. Cellular Nucleotide Pool and Its Regulation

**Figure 1:**
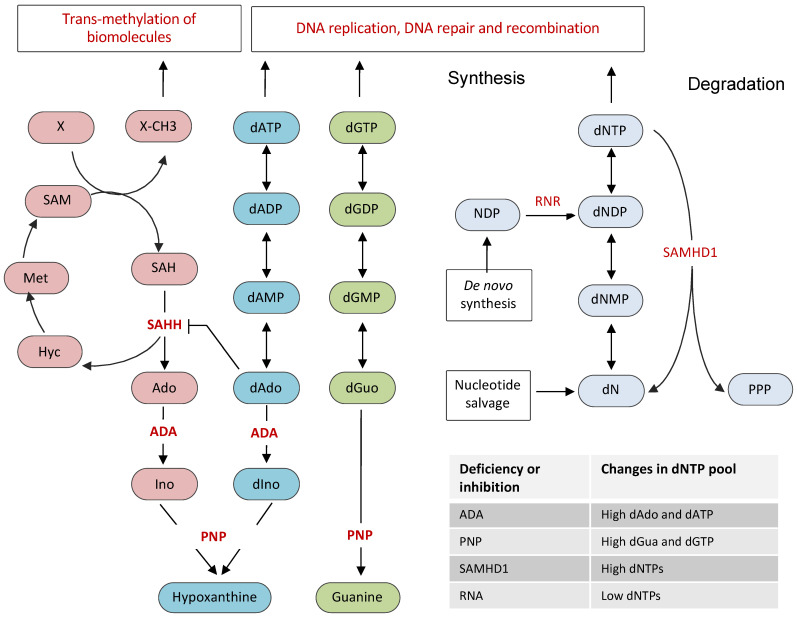
**Nucleotide metabolic pathways associated with antibody gene diversification.** The optimal dNTP pool is determined by the balance between de novo synthesis, consumption of synthesized nucleotides, and their degradation. RNR is the key enzyme that provides the balanced supply of dNTPs by converting ribonucleotides to deoxyribonucleotides. The nucleotide salvage pathways contribute to the dNTP pool by converting nitrogen bases and nucleosides back to nucleotides. SAMHD1, a dNTP hydrolase, degrades nucleotides to maintain low levels of dNTPs in the G1 phase. ADA and PNP are the key enzymes of the purine salvage pathway. ADA converts deoxyadenosine to deoxyinosine, and adenosine to inosine, respectively. PNP converts guanosine and deoxyguanosine to guanine, inosine, and deoxyinosine to hypoxanthine. The accumulated dAdo in ADA deficiency inhibits SAH hydrolase, leading to accumulation of SAH, which inhibits transmethylation of various biomolecules. The effects of the inhibition or deficiency of RNR, SAMHD1, ADA, and PNP are also summarized. RNR: ribonucleotide reductase; SAMHD1: sterile alpha motif and histidine-aspartic acid domain-containing protein 1; dNTP: deoxynucleoside triphosphate; NDP: nucleoside diphosphate; dNDP: deoxynucleoside diphosphate; dNMP: deoxynucleoside monophosphate; dATP: deoxyadenosine triphosphate; dGTP: deoxyguanosine triphosphate; dNs: deoxyribonucleosides; PPP: triphosphate; ADA: adenosine deaminase; PNP: purine nucleoside phosphorylase; Ado: adenosine; dAdo: deoxyadenosine; Ino: Inosine; dIno: deoxyinosine; dGua: deoxyguanosine; SAM: S-adenosyl methionine; SAH: S-adenosyl homocysteine; SAHH: SAH hydrolase; Hyc: homocysteine; Met: methionine; X: a biomolecule; and X-CH3: a methylated biomolecule.

The de novo biosynthesis of dNTPs involves the reduction of ribonucleoside diphosphates (NDPs) to deoxyribonucleoside diphosphates (dNDPs), which upon phosphorylation yield dNTPs. This rate-limiting reduction of NDP to dNDP is catalyzed by an allosteric enzyme ribonucleotide reductase (RNR), a hetero-tetrameric enzyme composed of two large α-subunits and two small β-subunits (Figure 1) [14]. The large subunit is encoded by the *RRM1* gene, whereas the two closely related isoforms of the small subunit are encoded by *RRM2* and *RRM2B* genes. The cellular levels of dNTPs highly fluctuate during the cell cycle, being highest in the S phase and lowest in the G1 phase. Consistently, the activity of RNR, which is tightly regulated by transcriptional, post-transcriptional, and allosteric controls, is restricted to S, G2, and M phases and is eliminated from the G1 phase of the cell cycle. The β-subunits contain iron-oxygen centers that are essential for the communication with catalytic sites on the α-subunits. In addition to NDP-binding catalytic site, the α-subunit also contains the allosteric activity site and the substrate specificity site. The binding of nucleotides to allosteric sites not only affects the activity of RNR but also regulates its substrate specificity, which is critical for the maintenance of nucleotide pool balance. The activity site responds to ATP or dATP to adjust the enzyme’s overall activity, whereas the specificity site responds to dATP, dGTP, and dTTP to adjust the specificity for the four NDP substrates to produce a balanced amount of all four dNTPs [15,16,17]. The binding of dATP inactivates RNR by converting an active hetero-tetramer to an inactive hetero-octamer, whereas the binding of ATP stimulates RNR activity for all four NDPs [15,16,17]. Although the *RRM1* subunit is constitutively expressed throughout the cell cycle, the expression of the *RRM2* subunit is highest during the S phase, which boosts the activity of RNR in S, G2, and M phases of the cell cycle [18,19].

In addition to de novo synthesis, nucleotide salvage pathways also contribute to the dNTP pool. Salvage pathways convert bases and nucleosides from nucleic acid degradation or exogenous sources back to nucleotides (Figure 1) [20]. Purines are salvaged through the action of adenine phosphoribosyltransferase (APRT) and hypoxanthine-guanine phosphoribosyltransferase (HGPRT). APRT and HGPRT adds phosphoribosyl pyrophosphate to adenine and guanine, resulting in the formation of adenine monophosphate (AMP) or guanine monophosphate (GMP), respectively. The addition of 2-deoxy-alpha-D-ribose 1-phosphate to thymine by thymidine phosphorylase leads to the formation of thymidine, which is converted to thymidine monophosphate (TMP) by thymidine kinase. The cytidine and deoxycytidine nucleosides are salvaged either through their deamination to uridine and deoxyuridine or by direct phosphorylation by uridine/cytidine kinase to cytidine monophosphate (CMP) or deoxycytidine monophosphate (dCMP).

Another protein, which has been shown to be critical for regulating the dNTP pool in mammalian cells, is the sterile alpha motif and histidine-aspartic acid domain-containing protein 1 (SAMHD1), a dNTP triphosphohydrolase that hydrolyzes nucleotide triphosphate into triphosphate and a nucleoside (Figure 1) [21,22]. SAMHD1 mediated degradation of dNTPs plays an essential role in maintaining low levels of dNTP pools outside the S phase of the cell cycle. In fact, SAMHD1 but not RNR is the principal regulator of dNTP levels in mammalian cells, as indicated by the dramatic accumulation of dNTP pools in SAMHD1 depleted cells [23,24,25]. SAMHD1 expression restricts viral infection by depleting cellular dNTPs, which are essential for viral reverse transcription and replication [26,27,28,29]. SAMHD1 mutations are associated with Aicardi–Goutières syndrome (AGS), a congenital neurodegenerative autoimmune disorder. In addition, SAMHD1 is frequently mutated in chronic lymphocytic leukemia (CLL) and colorectal cancers [30,31]. SAMHD1 was recently shown to play dNTPase-dependent and dNTPase-independent functions in DSB repair by NHEJ and homologous recombination, respectively [32,33,34]. Although SAMHD1 was proposed to possess a nuclease activity [35,36], subsequent studies showed that the nuclease activity of SAMHD1 was due to contaminants present in the SAMHD1 preparations [37,38,39]. Structurally, SAMDH1 possesses an N-terminal SAM domain, followed by a catalytic HD domain and a short C-terminal end. It has been shown that dGTP acts as a substrate as well as an allosteric activator of SAMDH1. SAMDH1 is also stimulated by GTP, a nucleotide that exists in higher amounts than dGTP in most cells [40].

## 3. Cellular Nucleotide Pool and V(D)J Recombination

The V(D)J recombination, which joins V_H_, D_H_, and J_H_ gene segments on the heavy chain, and V_L_ and J_L_ gene segments on the light chain, is a complex and intricate process that produces the DNA segments encoding V regions of immunoglobulins [1]. The V(D)J recombination is initiated by the products of recombination activating genes (RAG1/RAG2), which are expressed in developing lymphocytes, where they induce DSBs at conserved recombination signal sequences (RSS) [41,42,43]. Upon identifying and interacting with RSS, the RAG cuts these signal sequences and releases V, D, and J gene segments [44,45,46,47]. The RSS consists of a highly conserved heptameric sequence and nonameric sequences separated by poorly conserved 12–23 base pair (bp)-long spacer sequences [44]. While the heptameric sequence plays an essential role as a recognition element, the nonameric sequence is dispensable for recombination [48]. The length of the spacers at the RSS sequences at the 3′ end of the V_H_ segment and the 5′ end of the J_H_ is 23 bp, whereas the D_H_ fragment contains 12 bp long spacer at both the 5′and 3′ RSS. The 12/23 rule ensures that efficient recombination takes place between RSS with different spacer lengths, ensuring the joining of D_H_ with J_H_ and V_H_, and preventing the joining of V_H_ with J_H_ [49,50].

The V(D)J recombinase comprises two lymphoid-specific proteins, RAG1 and RAG2, which function with non-lymphoid-specific DNA bending or twisting factors HMG1A and HMG1B to induce DNA cleavage [42,51]. The RAG complex binds to RSS sequences that need to be recombined and creates nicks at the junction between the coding segment and the RSS to form a nicked paired complex [52,53]. The free 3’-hydroxyl groups produced as a result of nicking then initiate transesterification reactions on the phosphorous atoms in opposite DNA strands, resulting in the development of hairpins on the coding gene segments and blunt ends on the signal sequence end. The second phase of recombination involves numerous non-homologous end-joining DNA factors that assist in joining the coding DNA ends and is characterized by the loss or addition of extra nucleotides at the junctions, thus contributing to junctional diversity [54]. An Artemis endonuclease unwraps the hairpin produced by the cleavage of RAG protein. The off-center nicking of hairpins results in the formation of short palindromic sequences called P-nucleotides [55]. In addition to P-nucleotides, junctional diversity also results from the random addition of a small number of N-nucleotides by terminal deoxynucleotidyl transferase (TdT) [45,56].

The TdT belongs to X family polymerases that can incorporate nucleotides at the 3’-end of DNA irrespective of DNA templates [45,56]. The presence of GC-rich nucleotides in the N region sequences of Ig genes suggests a preference of TdT for dGTP and dCTP. Studies on the inherited diseases resulting from deficiencies of enzymes involved in nucleotide metabolism highlight the importance of nucleotide pool imbalance in V(D)J recombination. Deficiencies in adenosine deaminase (ADA) and purine nucleoside phosphorylase (PNP) result in dNTP pool imbalances that affect lymphocyte development [57]. ADA, an essential enzyme of the purine salvage pathways, converts deoxyadenosine to deoxyinosine and adenosine to inosine (Figure 1). The deficiency in ADA leads to severe depletion of T- and B-lymphocytes and impaired cellular and humoral immunity and dysregulation, such as severe combined immunodeficiency disease. The severe immune deficiency caused by ADA deficiency is due to the accumulation and downstream effects of adenosine, deoxyadenosine, and dATP. Accumulated dATP also leads to the depletion of other dNTPs by feedback inhibition of RNR, resulting in impaired DNA synthesis, replication, and repair. As ADA is highly expressed in lymphoid tissues, particularly the thymus, ADA deficiency severely affects thymocyte distribution and development [58]. However, exactly which stage of thymocyte development and differentiation is affected by ADA deficiency remains largely unknown. Although, unlike T-lymphocytes, early B-lymphocyte development is not disturbed by ADA deficiency, it affects B-lymphocyte distribution with severe B-cell lymphopenia and hypoglobulinaemia. As the formation of the germinal centers is severely affected in ADA deficiency, antigen-dependent development of B cells and B-cell memory generation is likely affected [59]. ADA-deficient mice showed the reduced proliferative ability of B lymphocytes and accumulated IgM antibodies with concomitant decrements in IgG.

**Figure 2:**
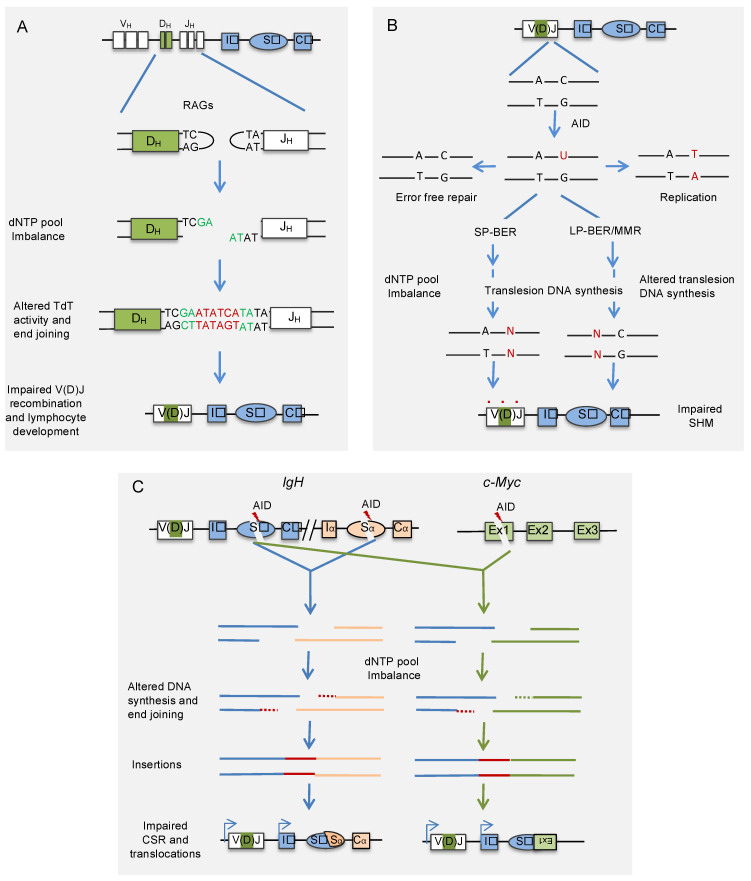
**Effect of nucleotide pool imbalance on antibody gene diversification.** (**A**) Imbalanced dNTP pool impairs the end-joining of RAG-induced DSBs by altering the activity of TdT, and possibly other DNA polymerases involved in end-joining during VDJ recombination. The red-colored nucleotides at the junction represent N-segment, and green-colored nucleotides represent P-segments. (**B**) The imbalance in the dNTP pool affects the translesion DNA polymerases involved in repairing mismatches produced by AID during hypermutation of recombined V region. (**C**) The dNTP pool imbalance also affects end-joining of AID-induced DSBs during physiological CSR and aberrant chromosomal translocations involving antibody genes. The imbalanced dNTP pool affects the activity of DNA polymerases and possibly other end-joining enzymes, leading to long insertions at the recombination junctions. AID: activation-induced cytidine deaminase; RAG: recombination activating genes; TdT: terminal deoxynucleotidyl transferase; SP-BER: short patch-base excision repair; LP-BER: long patch-base excision repair; MMR: mismatch repair.

Nucleotide pool imbalance due to ADA deficiency or supplementation of 2-deoxyadenosine in lymphoid cell lines transfected with V(D)J recombination substrates severely decreased V(D)J recombination frequency (Figure 2). Analysis of the recombination junctions revealed an increase in the insertions of A-T nucleotides at the coding joints, resulting in a two- to fourfold decrease in the ratio of G+C/A+T in the N regions [60]. Consistently, analysis of the N-region nucleotide compositions of V_H_-D_H_-J_H_ regions of Ig-μ heavy chains B-cells from ADA patients revealed an increase in A-T content, resulting in a ~threefold decrease in G+C/A+T ratios [60]. In addition, accumulated deoxyadenosine irreversibly inactivates S-adenosyl-L-homocysteine (SAH) hydrolase, leading to accumulation of SAH and inhibition of transmethylation, which is necessary for lymphocyte development (Figure 1) [61,62]. Because of the high proliferation rate of lymphocytes, ADA is highly expressed in lymphocytes, a possible reason for severe lymphotoxic effects of ADA deficiency.

PNP degrades guanosine and deoxyguanosine to guanine, and inosine and deoxyinosine to hypoxanthine. Like ADA, the deficiency of PNP results in the accumulation of deoxyguanosine, which gets phosphorylated to dGTP, thus resulting in its accumulation (Figure 1). Like the accumulation of dATP in ADA deficiency, the accumulated dGTP also exerts lymphotoxic effects with profound T-cell abnormalities and variable B-cell function [63,64]. The exposure of lymphoid cell lines transfected with V(D)J recombination substrates to deoxyguanosine does not affect the G–C content of N regions but leads to a fourfold decrease in the recombination of coding joints [60]. Although defects in the B-cell development in PNP deficiency are yet to be fully understood and are primarily attributed to T-cell abnormalities, PNP deficiency also has direct, T-cell independent effects on B-cell immunity [65]. The long-term exposure to hydroxyurea, an inhibitor of RNR, leads to an increase in illegitimate T-cell VDJ-recombination, suggesting that a deviation from optimal dNTP pool balance interferes with V(D)J recombination [66]. As SAMHD1 depletion leads to dramatic accumulation of both dATP and dGTP nucleotides, which interfere with SHM and CSR, it will be interesting to know if the B- and T-lymphocyte development and the process of V(D)J recombination is also affected by SAMHD1 depletion.

## 4. Cellular Nucleotide Pool and Somatic Hypermutation

An immunoglobulin is consists of a V region encoded by recombined V(D)J segments and a constant (C) region encoded by one of the Cμ, Cδ, Cα, Cε, or Cγ genes. The constant regions of all Igs, except Cδ, are preceded by highly repetitive switch (S) regions [1]. Both antigen-independent V(D)J recombination during early B-cell differentiation and antigen-mediated SHM and CSR in mature B cells are essential for antibody maturation. SHM, the driving force for antibody affinity maturation, introduces non-templated point mutations at a rate of 10^−2^–10^−3^ per base pair into the recombined variable regions (V) of both heavy and light chain Ig genes, resulting in the dramatic increments in the affinity of antibodies after affinity maturation in the germinal center of secondary lymphoid organs. On the other hand, CSR replaces heavy chain constant region exons encoding for the IgM with a downstream constant region encoding another antibody isotype, enabling B-cells to produce antibodies with diverse effector functions while retaining their antigen specificities.

Although mechanistically different, both SHM and CSR are initiated by AID-induced cytosine deamination of transcription-exposed single-stranded DNA, resulting in the formation of U/G mismatches and subsequent DNA cleavage in V or S regions, respectively [5,67,68]. While replication over U/G mismatches prior to the excision of uracil results in the transition of C:G to T:A, the vast majority of mutations during SHM are introduced by noncanonical G1 phase variants of base excision repair (BER) and mismatch repair (MMR) pathways, both of which employ error-prone DNA synthesis by translesion DNA polymerases [67,69,70,71]. While most U/G mismatches are faithfully repaired by canonical BER or MMR pathways, their error-prone repair by noncanonical BER or MMR pathways results in transition or transversion mutations at not only C/G but also flanking A/T base pairs. The excision of uracils primarily by UNG, and to a lesser extent, by SMUG1, the key enzymes of the BER pathway, generate abasic sites [72,73]. The repair of abasic sites by short-patch BER pathway, employing translesion DNA polymerases REV1 and Polη, generates transition and transversion mutations at G:C base pairs [73,74,75,76,77,78]. Alternatively, a long DNA patch surrounding U/G mismatches or abasic sites can also be excised by noncanonical MMR or long-patch BER, respectively. The stranded DNA gap produced thereby is filled by translesion DNA polymerases, resulting in transition and transversion mutations at A:T base pairs [79]. EXO1 creates the excision patches following nicking of the U/G mismatches or abasic sites by MutLα complex or apurinic/apyrimidinic endonucleases, respectively [79,80,81].

Interestingly, AID-induced SHM occurs in the G1 phase of the cell cycle, where dNTP pool concentrations are lowest [82,83]. The restriction of SHM to G1 phase suggests that dNTP paucity may promote SHM and that the accumulation of dNTPs or their imbalance may interfere with the normal functions of DNA polymerases involved in SHM. Recently, inactivation of *Samhd1* in mice carrying hen egg lysozyme (HEL) immunoglobulin-transgene (*SW_HEL_* mice) led to substantially increased levels of dNTPs in B cells [84]. Interestingly, this de-restriction of the dNTP pool in the G1 phase by the inactivation of *Samhd1* in germinal center B cells affected SHM (Figure 2). Lack of SAMHD1 resulted in a significant increase in C/G to A/T transition mutations, whereas transversions at A/T and C/G base pairs showed significant decreases. Similarly, an independent study also showed a decrease in the SHM frequency in the Sμ region of primary spleen cells derived from *Samhd1* KO mice [33]. These results suggest that SAMHD1-induced dNTP paucity contributes to AID-induced mutagenesis. Although, like *Samhd1* inactivation, *Msh2* knockout decreases A/T mutations and C/G transversions, the perturbation of SHM upon *Samhd1* inactivation was unlikely due to the inhibition of MMR. It is because *Samhd1* inactivation neither focuses on AID-induced mutation on AGCW hotspots nor shows strand bias, the signatures of MMR inactivation [85]. Similarly, it was also demonstrated that accumulation of dNTPs upon SAMHD1 inactivation does not alter the rate of uracil excision by inhibiting UNG activity [84]. The hypermutation phenotypes of *Samhd1* inactivated B cells show similarity with *PCNA*^−/−^ and *POLH*^−/−^ B cells. This observation suggests that the deficiency of SAMHD1 may reduce PCNA ubiquitination and Polη recruitment to V region of Ig without affecting the excision uracils. However, it remains to be analyzed whether dNTP pool imbalance caused by SAMHD1 deficiency impairs somatic hypermutation by affecting the recruitment of translesion DNA polymerases or modulating their activities. As SAMHD1 localizes to immunoglobulin genes in mouse B cell line, it would be interesting to analyze if it is implicated in the local depletion of dNTP pools to promote optimal SHM [33].

## 5. Cellular Nucleotide Pool and Class Switch Recombination

Generation of comprehensive humoral immune response requires high affinity and specific antibodies and the production of different antibody isotypes through CSR. The process of CSR changes the class or isotype of Ig expressed by B cells from IgM to other isotypes such as IgA, IgG, or IgE. Like SHM, AID-induced DNA breaks in Ig intronic switch (S) regions, positioned upstream of each of the constant regions of Ig isotype genes, initiates CSR [5,68]. CSR involves cleavage and joining of universal donor Sμ and one of the downstream acceptor S regions, which changes the constant region portion of the antibody heavy chain without affecting the variable region, and thus the antibody specificity remains unchanged (Figure 2). In CSR, the AID-induced SSBs in S regions are converted into DSBs, followed by the recombination of the two cleaved S regions by the action of classical non-homologous end-joining (c-NHEJ) or alternative end-joining (alt-EJ) pathways [67,69,86]. The joining of the cleaved S regions by c-NHEJ requires little or no sequence homology between cleaved S region DNA ends, whereas alt-EJ utilizes the microhomology (MH) between the single-stranded overhangs of the cleaved S region DNA ends. Usually, AID-induced DNA breaks are restricted to V or S regions of the Ig genes; however, uncontrolled expression or mistargeting of AID to non-Ig loci induces mutations and chromosomal translocations in both B lymphocytes as well as non-lymphocytic cells. The frequently observed chromosomal translocations between Ig locus and proto-oncogenes such as c-*MYC*, *FGFR*, *BCL-6*, or *BCL-2* are the hallmarks of B-cell malignancies [87,88,89,90].

In an attempt to identify proteins that function at AID-induced DNA-break sites in one of the S regions of the mouse Ig locus, a recent study applied locus-specific insertional chromatin immunoprecipitation and identified SAMHD1 as a novel regulator of CSR [33,91]. Dramatic accumulation of dNTPs, particularly purines, was observed in SAMHD1 depleted mouse B cell line CH12F3-2A and spleen B cells derived from *Samhd1* KO mice. The accumulation of dNTPs impaired not only AID-induced CSR but also aberrant chromosomal translocations between IgH and c-Myc loci (Figure 2). Site-directed mutational analysis of SAMHD1 further revealed that the effects of SAMHD1 depletion on CSR and IgH/c-Myc translocations were dependent on its dNTPase activity. The defects in CSR and IgH/cMyc translocations were not due to impaired AID-induced DNA breaks or synapsis between the two S-regions. The analysis of the CSR and IgH/cMyc breakpoint junctions revealed that a high frequency of longer nucleotide insertions is a notable feature in SAMHD1 deficiency, indicating that accumulation of nucleotides affects the end-joining step of DSBs (Figure 2).

Interestingly, CSR mediated by CRISPR/Cas9-induced staggered but not blunt DSBs is impaired by SAMHD1 depletion. It is essential to mention that most DNA breaks by AID are staggered, that require efficient end-processing prior to their joining by c-NHEJ or alt-EJ pathways [92]. Consistently, the supplementation of purine nucleosides into the culture medium severely affected both CSR and Igh/cMyc translocations. These results suggest that the intracellular dNTP concentration is an important regulator of antibody diversity and genomic instability. Multiple mechanisms, including altered activities of DNA polymerases and dNTP sensitive DNA end-processing enzymes at DSBs may be affected by imbalanced dNTP pool, resulting in the impaired CSR and chromosomal translocations. Consistently, the overexpression of TdT in *Samhd1* KO but not in WT CH12F3-2A B cell line reduced the frequency of CSR and increased the insertional events at CSR junctions. Since mature B cells do not express TdT, it can be envisaged that SAMHD1 inactivation would affect the TdT activity and V(D)J recombination. Both V(D)J recombination and CSR rely on NHEJ mediated repair pathways, where the dNTP pool plays a critical role. Similar to the defects in CSR and IgH/cMyc translocations, the elevated dNTP pools showed significant increases in both the frequency and length of insertions at the CRISPR/Cas9-induced translocation junctions and the repair of plasmid-based artificial DNA repair substrates [32,33]. These data strongly suggest a role of the dNTP pool in regulating cellular processes that depend on NHEJ. As SAMHD1 also localizes to DNA break sites [30,33,34], the possible role of SAMHD1 in local depletion of dNTP pool in DSB repair by non-homologous end-joining remains to be analyzed. Interestingly, SAMHD1 has also been reported to promote homologous recombination through its ability to interact with and recruit C-terminal binding protein interacting protein (CtIP) at the DSB site [34]. Therefore, it is yet to be analyzed whether SAMHD1 deficiency perturbs antibody generation in chicken B cells, which diversify Ig genes by a gene conversion that relies on homologous recombination [93].

## 6. Conclusions

Maintaining adequate levels and balance of dNTPs within the permissible physiological range is an important determinant of the accuracy and activity of DNA polymerases, which are involved not only in genomic DNA replication but also in DNA repair and recombination. The ability of the dNTP pool imbalance to modulate the rate and fidelity of DNA synthesis is strongly associated with the ability of the cell to protect against genomic instability induced by various environmental factors and physiological processes. The objective of this review was to discuss how dNTP pool balance affects the three critical processes involved in antibody gene diversification, namely V(D)J recombination, SHM, and CSR. While V(D)J recombination and CSR involve joining of RAG- or AID-induced DNA double-strand breaks, respectively, by NHEJ pathway proteins, SHM relies on BER and MMR of AID-induced uracils to introduce mutations into the recombined V region. In mammalian cells, the activity of NHEJ is maximum during the G1 phase of the cell cycle, where dNTP concentrations are lowest due to low RNR and high SAMHD1 activities. Interestingly, all three antibody diversification processes are predominantly restricted to the G1 phase of the cell cycle, indicating that antibody gene diversification has evolved to function at low concentrations of the dNTP pool [82,83,94] (Figure 2). Therefore, it is not surprising that the accumulation and imbalance of dNTPs interfere with antibody gene diversification processes.

The dNTPs are synthesized by RNR in the cytoplasm and diffused to the nucleus for DNA synthesis [95]. However, a number of reports showed that RNR is also localized to the sites of DNA breaks [96,97,98,99]. In contrast to RNR, SAMHD1 is mainly localized to the nucleus, which may be helpful in the robust depletion of the nuclear dNTP pool outside the S-phase of the cell cycle. Like RNR, SAMHD1 was found associated with chromatin at DNA break repair sites [33,34]. These findings suggest that the repair of DNA breaks may require local adjustment of dNTP concentrations for efficient DNA end-joining. However, the analysis of the local variations in the dNTP pool would require the development of advanced probes to measure local dNTP concentrations accurately.

The accumulation of purine nucleotides affects lymphocyte development at the stage of V(D)J recombination and causes severe immunodeficiencies [57]. Similarly, the accumulation of purine nucleotides caused by SAMHD1 deficiency impairs both SHM and CSR in mice and B-cell lines [33,84]. The purine nucleotides are also accumulated in Aicardi–Goutières syndrome with germline SAMHD1 mutations. However, the effect of SAMHD1 inactivating mutations on immune system diversification in patients with Aicardi–Goutières syndrome is yet to be analyzed. The imbalance in the dNTP pool affects V(D)J recombination and CSR by interfering with the end joining, whereas SHM is affected due to impaired translesion DNA synthesis. Although transition mutations are the most frequent mutations in our genome, they are less mutagenic than transversion mutations. The restriction of dNTPs by SAMHD1 promote transversion mutations during SHM, thereby affecting the binding characteristics of the antibodies. Therefore, it is conceivable to believe that patients with SAMHD1 inactivating mutations may be compromised in producing effective immune response. The imbalanced dNTP pool is known to increase the formation of mismatches and affect the accuracy of MMR [100]. For example, the accumulation of dNTPs in colon cancers with heterozygous SAMHD1 mutations exacerbate MMR mutation phenotypes [31]. Template-dependent DNA polymerases mu (μ) and lambda (λ) and template-independent TdT play non-overlapping roles in junctional processing during V(D)J recombination. Thus, imbalance in the dNTP pool may affect V(D)J recombination by modulating the activities of not only TdT but also polymerase μ and λ [101].

The accumulation of purine dNTPs leads to increased frequency of insertions at recombination junctions that are inhibitory for CSR and IgH/c-Myc translocations, suggesting that dNTP pool restriction favors DNA repair by NHEJ. Recent studies indicate that DNA polymerase theta (POLQ) is responsible for nucleotide insertions during NHEJ [102,103,104]. Interestingly, deficiency in POLQ leads to decreased insertions at the CSR junctions and increased frequency of IgH/c-Myc translocations [103]. These findings suggest that dNTP pool imbalance may modulate the activity of DNA polymerases involved in the end-joining. In fact, the accumulation of purine nucleotides leads to increased insertional events and reduced CSR activity in B-cell lines overexpressing TdT. An elevated dNTP pool may also affect end-resection during the end-joining of the DNA breaks. In addition, dNTP pool imbalance may also affect other dNTP-sensitive enzymes such as DNA/RNA helicases [105,106,107]. Long templated insertions have also been observed between the V and DJ segments in the human Ig locus and are associated with the development of effective antibodies [108]. Together, these findings suggest that dNTP imbalance can modulate the activities of DNA repair enzymes involved in antibody gene diversification.

## 7. Future Directions

Extrapolating from the defects observed in lymphocyte development and V(D)J recombination in patients with ADA and PNP deficiencies, it is conceivable that the dNTP pool imbalance caused by deficiencies of other enzymes of nucleotide metabolism may also affect V(D)J recombination. For instance, SAMHD1 could be playing an important role in maintaining the dNTP pool balance for optimal V(D)J recombination. Therefore, it is important to study if the restriction of the dNTP pool by SAMHD 1 during B-cell development affects the frequency and junctional diversity in V(D)J recombination. Similarly, it would also be important to study if ADA and PNP are required for AID-induced physiological and aberrant recombination. More importantly, further studies are also required to identify DNA polymerases primarily affected by dNTP pool imbalance caused by ADA, PNP, and SAMHD1 inactivation during V(D)J recombination, CSR, SHM, and the genomic instability associated with these processes. In case of ADA deficiency, modulation of the activity of TdT is likely responsible for the defects in V(D)J recombination. However, it remains to be analyzed if the imbalance in the dNTP pool also affects the activities of polymerase μ and λ which are implicated in V(D)J recombination. Similarly, the DNA polymerases whose activities are affected by dNTP de-restriction upon SAMHD1 depletion during AID-induced CSR, SHM, and chromosomal translocations are yet to be identified.
